# Ecology and Biogeography of Free-Living Nematodes Associated with Chemosynthetic Environments in the Deep Sea: A Review

**DOI:** 10.1371/journal.pone.0012449

**Published:** 2010-08-27

**Authors:** Ann Vanreusel, Annelies De Groote, Sabine Gollner, Monika Bright

**Affiliations:** 1 Marine Biology Research Group, Ghent University, Ghent, Belgium; 2 Department of Marine Biology, University of Vienna, Vienna, Austria; Northern Fisheries Centre, Australia

## Abstract

**Background:**

Here, insight is provided into the present knowledge on free-living nematodes associated with chemosynthetic environments in the deep sea. It was investigated if the same trends of high standing stock, low diversity, and the dominance of a specialized fauna, as observed for macro-invertebrates, are also present in the nematodes in both vents and seeps.

**Methodology:**

This review is based on existing literature, in combination with integrated analysis of datasets, obtained through the Census of Marine Life program on Biogeography of Deep-Water Chemosynthetic Ecosystems (ChEss).

**Findings:**

Nematodes are often thriving in the sulphidic sediments of deep cold seeps, with standing stock values ocassionaly exceeding largely the numbers at background sites. Vents seem not characterized by elevated densities. Both chemosynthetic driven ecosystems are showing low nematode diversity, and high dominance of single species. Genera richness seems inversely correlated to vent and seep fluid emissions, associated with distinct habitat types. Deep-sea cold seeps and hydrothermal vents are, however, highly dissimilar in terms of community composition and dominant taxa. There is no unique affinity of particular nematode taxa with seeps or vents.

**Conclusions:**

It seems that shallow water relatives, rather than typical deep-sea taxa, have successfully colonized the reduced sediments of seeps at large water depth. For vents, the taxonomic similarity with adjacent regular sediments is much higher, supporting rather the importance of local adaptation, than that of long distance distribution. Likely the ephemeral nature of vents, its long distance offshore and the absence of pelagic transport mechanisms, have prevented so far the establishment of a successful and typical vent nematode fauna. Some future perspectives in meiofauna research are provided in order to get a more integrated picture of vent and seep biological processes, including all components of the marine ecosystem.

## Introduction

Light in the deep sea only penetrates in the upper layers of the water column, so that photosynthesis is limited to a few 100 meters water depth. The main energy source for deep-sea communities is derived from this surface primary production. However, through its long transport from the surface, much of the photosynthetic derived material is mineralized, before reaching the deep-sea floor. This lower energetic input results in general in a significant decrease in standing stock of benthic communities, from the shelf along the continental slope towards the abyss [1]. The local diversity, in contrast, is in general high on the deep-sea floor, although the true extent of regional or global biodiversity is still unknown [1]–[3].

Remarkable exceptions on these general trends of high local diversity and low density and biomass are ecosystems, known as hydrothermal vents [4], and cold seeps [5]. These systems are largely driven by chemosynthetic derived energy, and not directly dependent on photosynthesis. They appear locally on active or passive margins in the case of cold seeps, or mid-ocean ridges and back-arc basins in the case of hydrothermal vents [e.g.4]–[7]. Both systems share common characteristics such as the presence of reduced chemical compounds (H_2_S and hydrocarbonates), local hypoxia or even anoxia, a high abundance and metabolic activity of bacterial populations, and the production of autochthonous, organic material by chemoautotrophic bacteria. However, hydrothermal vents and cold seeps differ also in many ways. Compared to the more stable cold seeps, vents are characterized by locally high temperatures, strongly fluctuating temperatures, pH, sulphide and oxygen concentrations, often the absence of sediments, a relatively young age, and often unpredictable conditions, such as waxing and waning of vent fluids or volcanic eruptions [4].

Despite the extreme physico-chemical conditions, compared to the surrounding seafloor, several taxa survive, or even thrive in these environments [8]–[14]. Both hydrothermal vents and cold seeps show regularly, highly increased levels of metazoan biomass [15], [5], in association with a low local diversity. This is explained through the presence of dense aggregations of foundation species and epizooic animals, living within these aggregations. Although the importance of chemosynthesis in the deep sea has been known for several decennia already (hydrothermal vents were discovered in 1977, cold seeps in 1984) [16], it is only relatively recently, through the more general use of Remote Operated Vehicles and submersibles, that more insight has been gained into specific interactions between seep and vent fauna, and their reduced environments. However biological research in cold seeps and hydrothermal vents has been mostly focused on the microbiology [4], [17], [18], and the prominent chemosynthetic macro-invertebrates [5], [19], [20]. Much less research has been done on the smaller benthic fraction at the size of the meiofauna (<1 mm), with as dominant taxon, the nematodes.

Nematodes are among the most abundant metazoan taxa in deep-sea habitats in general [21]–[24]. They are considered as important indicators of habitat heterogeneity in marine environments, including the deep sea, since they are common, numerous and speciose, and in close contact with seafloor related processes [3]. Despite their numerical importance, still little is known on their ecology and distribution in the deep sea, especially in association with seeps and vents.

Through a review of existing literature, and in combination with an integrated analysis of datasets obtained through the Census of Marine Life program on Biogeography of Deep-Water Chemosynthetic Ecosystems (ChEss), insight is provided into the taxonomy, ecology and biogeography of free-living nematodes, associated with chemosynthetic environments in the deep sea. Inevitably, the compilation of various datasets, collected by different researchers, includes a high degree of heterogeneity, partly generated by differences in temporal and spatial scales of sampling. Furthermore, the sampling design is highly unbalanced, leading to underrepresentation of different habitats and regions. Therefore, caution is needed in the interpretation of the results, considering the fragmented nature of the observations (see also [3]). In this review four main objectives were put forward, taking into account the restrictions of the dataset: 1) we investigated the extent to which the same trends of high standing stock and low diversity, as observed for many macro-invertebrates, were also present for nematodes in both vents and seeps; 2) because of the extreme conditions of low oxygen and high sulphide concentrations, and in case of vents additonally temperature fluctuations, it was further explored to which degree the present nematode fauna at vents and seeps differs from the regular deep-sea fauna; 3) related to the previous objective we also compared if the present taxa and communities showed respectively similar adaptations and composition in both vents and seeps; and finally 4) we examined the degree of connectivity between isolated chemosynthetic-driven sites in the deep sea.

## Materials and Methods

A total of 36 studies examined nematode abundances from chemosynthetic habitats, 21 of them were carried out in the deep sea, and 15 in shallow waters (<200 meters). Deep-sea cold seeps are represented by 11 studies [8], [9], [12], [25]–[32], deep-sea hydrothermal vents by 9 studies [33]–[41], and one study is available on deep-sea whale falls [42]. Nine studies were performed in shallow-water seeps [43]–[51], and 6 in shallow-water vents [52]–[57]. In order to give a complete overview in this review, all abundance data are listed in Table 1, 2, 3 and 4. For some studies we had to recalculate data, in order to get standardized nematode abundances per 10 cm^2^ (i.e. from given total meiofauna abundances found at a certain surface area, and given relative nematode abundances, we recalculated nematode abundance per 10 cm^2^) [12], [40], [49]. In one study, abundances were estimated from nematode biovolumes [9], and in another one, we estimated abundances from given figures in the manuscript [25]. We tried to standardize data to 10 cm^−2^, whenever it was possible, but for a few studies we had to give abundance data per 10 cm^−3^. In all of these studies it became obvious that nematodes are mostly one of the dominant meiobenthic taxa.

**Table 1:** Meiofauna at seeps and vents.

	Type	Depth (m)	Sampling technique	Habitat	Abundance (10 cm^2^)	Reference
**Shallow cold seep**						
Isla Vista, Santa Barbara Channel	oil/gas	15	corer	bacterial mats	326–3070	46
Isla Vista, Santa Barbara Channel	oil/gas	18	corer	bacterial mats	mean 1310–2420	47
Isla Vista, Santa Barbara Channel	oil/gas	18	corer	bacterial mats	*1573–2866 (10 cm^3^)*	48
Isla Vista, Santa Barbara Channel	oil/gas	19	corer	bacterial mats	∼2000	29
East Flower Garden, Gulf of Mexico	Brine seep	72	scoop	bacterial mats	*1 to 82 (10 cm^3^)*	49
East Flower Garden, Gulf of Mexico	Brine seep	72	grab	bacterial mats	1 to 23	50
Dnieper Canyon, Black Sea	gas	182–252	corer	bacterial mats	1 to 29	51
**Deep cold seep**						
Hydrate Ridge, off Oregon	gas hydrate	800	corer	bacterial mats	80–213	32
	gas hydrate	800	corer	underneath clams	626–467	32
Monterey Bay, off California	Gas	906	corer	n/a	9–307	9
Blake Ridge, Atlantic	Gas	2154–2158	corer	bacterial mats	*2 to 55 (10 cm^3^)*	12
	gas	2155–2157	corer	underneath bivalves	*41–78 (10 cm^3^)*	12
	gas	2157	corer	underneath xenophyophore	*18–23 (10 cm^3^)*	12
AC-AV-GC*, Gulf of Mexico	gas	692–2238	corer	bacterial mats	*108–4809 (10 cm^3^)*	12
Häkon Mosby, Barents Sea	Mud volcano	1288	corer	reduced sediments	2381	31
	Mud volcano	1287–1294	corer	Sclerolinum	1633–2728	31
	Mud volcano	1288	corer	bacterial mats	2798	31
Barbados Trench, Caribbean Sea	gas	5000	corer	reduced sediments	116	8
	gas	5000	corer	underneath bivalves	6505–8336	8
AC-AV-GC*, Gulf of Mexico	gas	1400–2800	Bushmaster Jr.	ass. Lamellibrachia	1 to 447	28
**Shallow vent**						
Bay of Plenty, New Zealand	vent	8 to 11	corer	bacterial mats	1 to 211	53
Matupi Harbour, Papua New Guinea	vent	0 to 27	corer	bacterial mats	*1 to 131 (10 cm^3^)*	54
**Deep whale falls**						
Santa Cruz Basin, off California	whale fall	1675	corer	0–3 m from whale bones	41±25	42

Overview of nematode abundance data from deep and shallow cold seeps, hydrothermal vents and whale falls. For seeps and vents only studies at higher taxonomic level are included.

**Table 2:** Nematodes at deep cold seeps.

Location	Type	Depth (m)	Sampling technique	Habitat	Abundance (10 cm^2^)	Nematode details	Reference
Hatsushima, Sagami Bay – **cd 1**	gas	1100	corer	underneath clams	207–384	*Daptonema*, *Chromadorita*	30
Häkon Mosby, Barents Sea - **cd 2**	Mud volcano	1286	MUC	sediment centre	22.6±24.9	*Halomonhystera disjuncta*	25
	Mud volcano	1288	MUC	Siboglinidae field	1575.4±564.6	*Monhystera*	25
	Mud volcano	1287	MUC	bacterial mats	11109.3±2267.9	*Halomonhystera disjuncta*	25
Nyegga, Norwegian margin - **cd3**	Pockmark	733	Push core	Siboglinidae field	6590.6±1098.9	*Aponema*, *Terschellingia*	26
	Pockmark	733	Push core	Black sediments	287.7±26.6	*Terschellingia*	26
Storegga, Norwegian margin - **cd4**	Pockmark	746	Push core	Siboglinidae field	39.1±20.6	*Sabatieria*, *Rhabdocoma*	26
Häkon Mosby, Barents Sea - **cd2**	Mud volcano	1255	Push core	Grey bacterial mats	1137±693.8	*Halomonhystera disjuncta*	26
REGAB, Gulf of Guinea,- **cd5**	Pockmark	3150	Push core	Clam-Mussel patch	9.9–842.7	*Sabatieria mortenseni*	27
						*Desmodora*	

Overview of nematode abundance and dominant species/genera data from deep cold seeps.

**Table 3:** Nematodes at deep hydrothermal vents.

Location	Type	Depth (m)	Sampling technique	Habitat	Abundance (10 cm2)	Nematode details	Reference
North Fiji Basin,	Vent	1984–1993	grab	*Bathymodiolus* fields	3 42 (10 cm3)	*Monhystera*, *Leptolaimus*,,	37
NE Pacific – **hd 1**						*Molgolaimus*, *Marylynnia*,	
						*Acantholaimus*, *Desmodora*	
Snake Pit	Vent	3492	mussel pot	*Bathymodiolus* fields	28	*Thalassomonhystera*	38
Mid Atlantic Ridge -**hd 2**							
Buckfield	Vent	2480	mussel pot	*Bathymodiolus* fields	1–2	*Thalassomonhystera*	38
N East Pacific Rise - **hd 3**							
Buckfield	Vent	2480	mussel pot	*Bathymodiolus* fields	1–2	*Thalassomonhystera fisheri*	39
N East Pacific Rise - **hd 3**							
Riftia,	vent	2500	Bushmaster Jr.	*Riftia pachyptila*	<1–7	*Thalassomonhystera fisheri*	39,35
N East Pacific Rise - **hd 4**							
Tica, N East Pacific Rise - **hd 5**	vent	2500	Bushmaster Jr.	*Riftia pachyptila*	<16–946	*Thalassomonhystera fisheri*	39,35
Biovent, East Wall, Train Staion, N	vent	±2494	Mussel pot	*Bathymodiolus* fields	22–116	*Thalassomonhystera*	33
East Pacific Rise - **hd 6–8**							
Rehu Marka, Oasis,	vent	2581–2690	Mussel pot	*Bathymodiolus* fields	50–72	*Thalassomonhystera*, *Anticoma*	33
Animal Farm, Buddha's Place							
S East Pacific Rise - **hd 9–12**							
N East Pacific Rise – **hd 7–8**	vent	2491–2690	mussel pot	*Bathymodiolus* fields	51.3	*Halomonhystera*,	34
						*Thalassomonhystera*	
Iheya Ridge Area, NE Pacific- **hd 13**	vent	1393	n/a	*Bathymodiolus* l fields	n/a	*Neochromadora*	41
Guaymas, East Pacific Rise	vent	1800–2600	n/a	Bacterial mats	1–78	*Desmodoridae*	40
Explorer Ridge - **hd 14–16**							

Overview of nematode abundance and dominant species/genera data from deep hydrothermal vents.

**Table 4:** Shallow seeps and hydrothermal vents.

Location	Type	Depth (m)	Sampling technique	Habitat	Abundance (10 cm2)	Nematode details	Reference
North Sea Pockmark	methane seep	153–167	box corer	reduced sediments	32.3–719.1	*Astomonema southwardorum*	43
East Flower Garden	brine seep	72	Grab sampler	bacterial mats	from Powell *et al.* 1983	*Monhystera anoxybiotica*,	44
NW Gulf of Mexico						*Gonionchus intermedius*,	
						*Linhomoeus gittingsi*,	
						*Mesacanthoides fibulatus*,	
						*Desmolaimoides thiobioticus*	
Kattegat, Denmark	“Bubbling reefs”	10–12	corer	reduced sediments	n/a	*Sabatieria punctata*, *Daptonema*,	45
	methane seep					*Leptonemella aphanotecae*	
Jan-Mayen ridge	vent	100–106	Slurp-gun	bacerial mats	n/a	*Linhomoeus aff. hirsutus*,	52
Subpolar, Mid Atlantic Ridge						*Desmodora scaldensis*,	
						*D. communis*,	
						*Anticoma acuminata*	
						*Enoplus communis*,	
						*Neochromadora poecilosoma*	
Milos, Mediterranean	vent	5–10	Push core	central sample	0 (10 cm^3^)	*Oncholaimus camplyloceroides*	55
	vent	5–10	Push core	bacterial mats	0–∼4 (10 cm^3^)	*Oncholaimus camplyloceroides*	55
	vent	5–10	Push core	edge of bacterial mats	∼10–∼36 (10 cm^3^)	*Oncholaimus camplyloceroides*	55
Milos, Mediterranean	vent	10	corer	White bacterial mats	0–1075	*Oncholaimus camplyloceroides*,	56
						*Chromadorina*, *Sabatieria*	
Sulawesi, Indonesia,	vent	3	corer	reduced sediments	25.15±12.25	*Pomponema*, *Dichromadora*,	57
equatorial Pacific				10 cm distance		*Oncholaimus*	
	vent	3	corer	reduced sediments	148.63±56.42	*Pomponema*, *Dichromadora*	57
				100 cm distance			

Overview of nematode abundance and dominant species/genera data from shallow cold seeps and hydrothermal vents.

**Figure 1:**
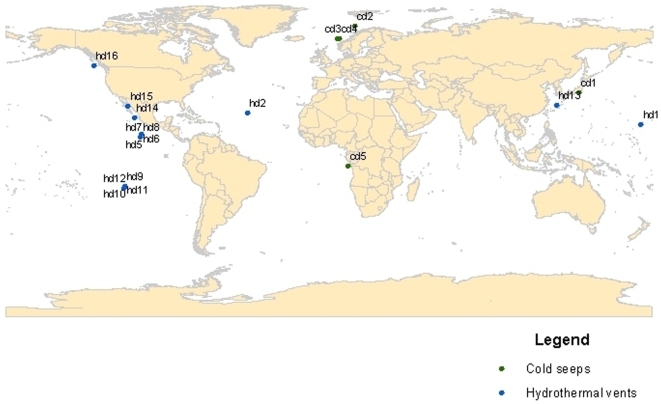
World map with indication of study sites on nematodes taxonomic composition from deep-sea vents and seeps.

Although many of these studies provide certain information on nematode families or genera, only 12 of them give complete genera or species abundance data (1 each on shallow-water vents and seeps [43], [57], 3 on deep-sea seeps [25]–[27], 7 on deep-sea vents [33]–[39]). Figure 1 and Tables 2 and 3 show the geographical distribution of the investigated deep-sea seeps and vents. We used data from these deep-sea chemosynthetic studies (including their controls) in order to evaluate univariate diversity measurements such as nematode genera richness (S), Shannon diversity index (H′), and Pielou's evenness (J) (all based on standardized genera richness data). Observed genera richness for deep-sea seeps, vents, and controls were plotted, using permuted sample-based rarefraction curves (Primerv6), that account for the patchiness in the data, resulting from natural sample heterogeneity. Student's t-test was performed to evaluate possible differences in univariate measurements of different ecosystems and habitat types. For multivariate analyses, we used Primerv6, in order to calculate similarity and dissimilarity of nematode communities from various ecosystems and habitats, using Bray-Curtis similarity (data were prior standardized and square-root transformed to down-weight the importance of very abundant species without losing the influence of rarer species). In addition, analysis of similarity (ANOSIM), and multi-dimensional scaling (MDS) were carried out with the same program.

## Results and Discussion

### Nematode standing stock at seeps and vents

Nematode densities and biomass along the regular slope generally decrease with water depth, surface primary productivity and distance offshore [58], [59]. They range between 10 individuals per 10 cm^2^ in the most oligotrophic seas, such as the central Arctic [23] or the East Mediterranean [60], to a maximum of several 1000 individuals per10 cm^2^ in the most productive areas such as the Weddell sea [61], or at other upwelling areas near oxygen minimum zones [62]. At abyssal plains surface productivity is mostly the main driving factor for nematode densities, often not exceeding 1000 ind. 10 cm^−2^ in the most eutrophic areas [63], [64], except at the base of canyons, which can serve as fast conduits of large quantities of organic material from terrigeneous origin [27], [65]. It is expected that the nematode standing stock at deep-sea seeps and vents is also depending on the availability of food, suggesting that the *in situ* chemosynthetic production may lead to elevated nematode densities and/or biomass compared to the adjacent phytodetritus fed sediments.

Seeps show a high variation in total densities, ocassionally amounting to several thousands of individuals per 10 cm^2^ (Table 1 and 2.). It illustrates that nematodes can benefit from the increased bacterial production at seeps by increasing their numbers. Ten fold higher densities were observed at several seep sites, compared to adjacent control sites [8], [25]–[27]. Nematode infauna densities in bivalve habitats range from ∼10 to ∼8300 ind. 10 cm^−2^ [8], [27], [30], [32], in the Siboglinid tube worm habitat from ∼40 to ∼6600 ind. 10 cm^−2^ [25], [26], [31], in bacterial mats from ∼80 to ∼11100 ind. 10 cm^−2^ [25], [26], [31], [32], and in other reduced sediments from ∼20 to ∼2400 ind. 10 cm^−2^ [8], [25], [26], [31]. Interestingly, epizooic nematode density at seeps from the Gulf of Mexico, associated with *Lamillibrachia* tubeworm and bivalve aggregations was very low ranging from 1 to 447 ind. 10 cm^−2^ [28]. It is unclear which factors are responsible for this high variation in nematode standing stock, but likely a combination of food availability, associated with seep intensity, but also the presence of soft substrates, are the main drivers. Toxicity does not seem to be a hampering factor, since the highest sulphide concentrations in deeper bacterial mat sediment layers at the Häkon Mosby Mud Volcano (HMMV, Barents sea slope) attained the highest densities (>10 000 ind. 10 cm^−2^). However, also the presence of other benthic taxa may interfer with the nematode success. In addition to nematodes, polychaetes of sulphide tolerant families, such as Capitellidae, Spionidae and Dorvilleidae, may thrive in the same seep conditions [5], and may compete with them for food, space or oxygen, or act as potential predators. Two studies indicate a significant negative correlation between meio- and macrofauna densities in sulphidic sediments from a whale fall [42] or at the seeps along the Nordic margin [26].

Increased standing stock is not only explained by increased densities. Some studies [37], [44] found that longer nematodes dominate in cold seep and hydrothermal sediments, compared to oxic neighboring sites. In [37], nematodes present in the hydrothermal vent are on average twice as large (800 µm long, 20 µm width), as those in the reference sediment (480 µm long, 15 µm width). Also the REGAB seep at the Guinea basin shows much heavier nematodes in the seep (0.32–0.94 µg DW), compared to the control sediments (0.03–0.18 µg DW) [27]. Jensen [44], [66] already pointed to a significant difference in body shape between oxybiotic (surface-dweller) and thiobiotic (deeper-living) nematodes in two subtidal sediments, and suggested that the pronounced body elongation,and the suggested increase in surface-volume ratio in thiobiotic species, is an adaptive character related to low oxygen partial pressure, and epidermal uptake of dissolved organic matter as additional nourishment of thiobiotic species [67]. [68] suggests, that the trend of increased length in suboxic or anoxic conditions, reflects an increased mobility. Nematodes are rather tolerant to anoxia, but they do not survive long-term exposure [69]. As nematodes respire aerobically, they cannot be permanent resident in anoxic sediments and, to avoid damaging conditions associated with long-term exposure to anoxia, they need the capacity to move away [66], [70], [71].

In contrast to seeps, deep-water hydrothermal vents in general do not show high nematode densities or biomass. The communities are often impoverished or show similar densities compared to their adjacent background habitats [33]–[39], [41] (Table 1 and 3). In bacterial mats 1–78 ind. 10 cm^−2^ [40] occurred, and on bivalves also only 1 to 72 ind.10 cm^−2^ were found [33], [34], [38], [39]. The tube worm habitat showed a high variation from very low nematode densities (1 ind. 10 cm^−2^) to higher values of about 900 ind. 10 cm^−2^ [35]. It illustrates a high patchiness, definitely supporting the need for more studies at vents based on replicated sampling designs.

The general observation of low nematode densities at vents so far, is rather striking and at first sight controversial: energy and space are copious and plentious in a harsh, although inhabitable biochemical environment. Three possible reasons for the low nematode densities at vents are put forward: (1) The substrate is unsuitable for supporting high abundances. Vents consist of recently formed hard substrates, such as basalt or sulphide minerals precipitates, with no or little sediment covering the hard substrate. Vent fluid reaches the water column through sulphide chimneys or crack and crevices of basalt, on which large foundation species, such as bivalves and tubeworms, grow. Most hydrothermal vents provide living space for meiofauna only on these hard substrates, or on the associated foundation species. However, overall nematode communities are more abundant in sediments than in epibenthic or epizooic/epiphytal communities, [72]. Also in other hard substrate, deep-sea habitats such as *Lophelia* coral rubble [73] or abyssal manganese nodules [74], nematodes are regular members of the so-called ‘aufwuchs’ communities, but occur as well in low abundances (i.e. only maximal 160 specimens per nodule with a diameters of 10 to 16 cm). In contrast, at cold seeps pore water with reduced gasses percolates to the seafloor surface through the soft sediment, in which the meiofauna lives. This supports the idea, that sediment offers a better potential living space for nematodes than hard substrate [75], allowing higher nematode abundances in seeps, than in vents which are mainly lacking soft sediments. Only one study sofar, also looked at epizooic fauna from seeps, and recorded the same low densities as at vents [28].

(2) Bottom-up control, where increased energy input is not available as food for the present nematodes. Although primary production is high at hydrothermal vents, and most nematode species known from vents are considered as deposit- and bacteria-feeders [39], it is unknown yet if the quality of food can in principal sustain high nematode populations at vents. So far no trophic studies were performed on vent nematodes.

(3) Top-down control, where the numbers of nematodes are kept low due to biotic interactions such as predation or competition. It is known that vent macro-invertebrates can occur in extremly high abundances [4], [76]. In what way nematodes interfer with these other, much larger organisms, but also with other meiofauna such as copepods, is unclear yet.

### Nematode diversity at seeps and vents

**Figure 2:**
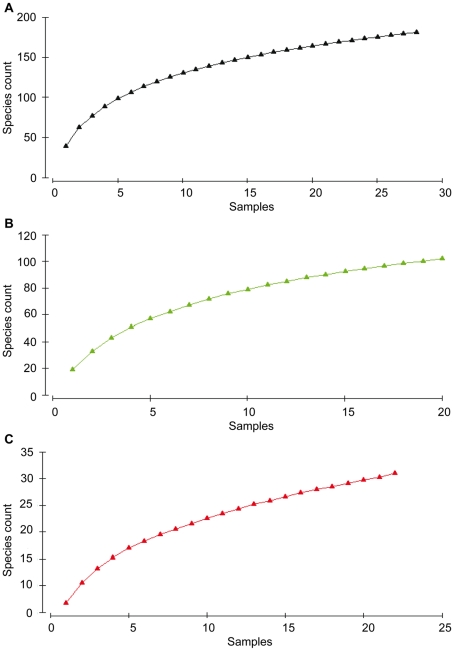
Permuted sample-based rarefraction curves for observed genera in deep-sea control samples: A. 27 samples (black colour code), cold seeps ; B. 20 samples (green colour code), and hydrothermal vents; C. 22 samples (red colour code).

Local diversity of the benthos is in general relatively high in deep-sea sediments, with low dominance and a high number of species co-existing on the same spot [2], [77]. For nematodes it is not different: the most abundant or dominant genera, like *Acantholiamus*, *Thalassomonhystera*, *Microlaimus* or *Leptolaimus*, represent the communities for less than 20% in general, and contain many con-generic species [3 and references therein]. Local species numbers can reach values higher than 100, whereas species richness often increases with densities [78]–[80]. Diversity of the here presented control sediments (mean S: 44; J′: 0.8; H′_loge_: 2.8; with S standing for genera counts) was significantly higher, compared to seeps (mean S: 18; J′: 0.5; H′_loge_: 1.4). A similar, but less pronounced nematode diversity pattern was also observed for hard substrate control sites (mean S: 10; J′: 0.9; H′_loge_: 2.1) and hard substrate vent sites (mean S: 7; J′: 0.6; H′_loge_: 1.0) (Tables 5 and 6). Sample-based rarefraction curves showed that observed genera richness after analysing 20 samples, was very high at deep-sea control samples (S: 165), intermediate at seeps (S: 102) and lowest at vents (S: 30) (Fig. 2). The low diversity of free-living nematodes at both seeps and vents is therefore in strong contrast to their surroundings.

**Table 5:** Mean nematode genera richness (S), standard deviation of genera richness (S stdv), Pielou's evenness (J′) and its standard deviation, and Shannon diversity index (H′_loge_) and its standard deviation are presented for all yet available data from deep-sea cold seeps (seep) and hydrothermal vents (vent).

	S	S (stdv)	J**′**	J**′** (stdv)	H**′**_loge_	H**′**_loge_(stdv)	sim. (%)
Seep	**18.25**	16.08	**0.49**	0.28	**1.41**	1.05	21
Control seep	**43.7**	14.42	**0.78**	0.07	**2.81**	0.49	44
Vent	**6.82**	3.45	**0.6**	0.23	**1.03**	0.61	49
Control vent basalt	**10**	1.83	**0.9**	0.1	**2.05**	0.25	41
Control vent sediment	**43.5**	10.61	**0.68**	0.01	**2.57**	0.21	72
Epifauna Chem. H.	**6.25**	3.06	**0.59**	0.23	**0.96**	0.56	41
Infauna Chem. H.	**17.73**	15.38	**0.51**	0.28	**1.43**	1.02	19
Vent-Gradient:							
V-pompei worms	**0**	0	**0**	0	**0**	0	0
V-vestimentiferans	**2.17**	0.75	**0.6**	0.31	**0.35**	0.23	63
V-bivalves	**8.56**	2.13	**0.6**	0.21	**1.28**	0.5	57
Seep-Gradient:							
S-reduced sediments	**11.2**	4.66	**0.45**	0.19	**1.09**	0.62	23
S-bacterial mats	**8.67**	8.33	**0.02**	0.02	**0.03**	0.01	92
S-Sclerolinum	**28.75**	20.61	**0.76**	0.11	**2.39**	0.63	38
S-bivalves	**8**	3.24	**0.45**	0.16	**0.9**	0.35	48

Information is also given for control samples (control) (no direct influence of chemosysnthesis), epi- and infauna of chemosynthetic habitats (Chem. H.), and various habitat types of vents and seeps. In adddition to the univariate diversity measurments, similarity (sim. %) based on Bray Curtis similarity is given.

**Table 6:** Results of student's t-test (p) are given for genera richness (S), Pielou's evenness (J′), and Shannon diversity index (H′_loge_).

	p (S)	p (J**′**)	p (H**′**loge)	dissim. (%)	R	p (Anosim)
Vent - Seep	**<0.01**	0.18	0.16	91	0.63	**0.001**
Vent - Control vent basalt	0.10	**0.02**	**<0.01**	75	0.75	**0.002**
Seep - Control seep	**<0.01**	**<0.01**	**<0.01**	78	0.26	**0.001**
Infauna - Epifauna Chem. H.	**<0.01**	0.31	0.07	87	0.39	**0.001**
Vent-habitats						
Vestimentiferans - bivalves	**<0.01**	0.99	**<0.01**	61	0.55	**0.001**
Seep-habitats						
Red. sed. - bac. mats	0.49	**0.01**	**0.02**	63	0.15	0.160
Red. Sed. - *Sclerolinum*	0.09	**0.02**	**0.02**	83	0.54	**0.003**
Red. Sed. - bivalves	0.27	0.98	0.56	90	0.59	**0.008**
Bac. mats - *Sclerolinum*	0.13	**<0.01**	**<0.01**	97	1	**0.008**
Bac. mats - bivalves	0.91	**<0.01**	**<0.01**	97	1	**0.018**
*Sclerolinum* - bivalves	0.04	**0.01**	**<0.01**	86	0.88	0.100

In addition, dissimilarity (dissim.) (%), based on Bray Curtis similarity, and Anosim's R and p are presented to show significant differences (p values<0.05 are marked in bold) of different ecosystems and habitats.

Nematode genera richness is in general low (18±16) in the seep ecosystem, and there is no statistically discernable difference in genera richness between various habitat types, such as bare reduced sediments (11±5), bacterial mats (9±8), Siboglinid fields (29±21), or bivalve fields (8±3) (Tables 5, 6; data from [25]–[27]). Also, Shannon diversity indices and Pielou's evenness are overall low at seeps (J′: 0.5±0.3; H′_loge_: 1.4±1.1). Seep sediments are often dominated by a single species, representing 50 to 90% of the total community. However, the number of other nematode taxa still can be relatively high at seeps, but each of these taxa are represented by only a few individuals per 10 cm^2^. Only Siboglinid fields are an exception, with the dominant genus being represented by only 12 to 49%. Interestingly, lowest evenness and lowest diversity are detected in bacterial mats (mean J′: 0.02; H′_loge_: 0.03). In this habitat the oxygen layer was only 1 mm thin, and total sulphide concentrations were up to 1 mMol. Intermediate values are present in bare reduced sediments (mean J′: 0.5; H′_loge_: 1.1) and bivalve fields (mean J′: 0.5; H′_loge_: 0.9). The reduced sediments (intermediate diversity) in the center of the HMMV were charcterized by a 1–3 mm thin oxydized sediment layer, slighlty elevated temperatures, but no sulphide concentrations [81]. No environmental data were available for bivalves. Highest evenness and diversity was noticed in Siboglinid fields (mean J′: 0.8; H′_loge_: 2.4) (Tables 5 and 6), where 3 to 10 cm of the sediments were oxic [25], [81].

The low genera richness, low diversity and low evenness points to the fact that the additional chemical energy source at seeps stimulates only a few species, that respond significantly to the increased food availability. There are two possible explanations for the low diversity in association with often (but not always) high densities at seeps: (1) only single species from the deep sea are adaptated to the toxic environment, and (2) the opportunistic behaviour of seep thriving nematode species results in competitive exclusion of other species; the first possibly explaining the lower number of species, the second explaining the low eveness and high dominance. Since several species are present at seeps, it is likely that the higher food input results in a competitive advantage of fast growing species that are blooming, whereas others with slower growth remain at constant low levels.

Vents are also characterized by low diverse and uneven nematode communities (S: 7±3; J′: 0.6±0.2; H′_loge_: 1.0±0.6) (data from [33], [35]–[39]). Interestingly, vents show a clear pattern of genera richness and diversity inverse correlated to stress (vent fluid emissions). In the high-flow Pompei worm habitat, with highly changing temperatures ranging from 14 to 119°C, high sulphide concentrations (e.g. >1 mM), and low pH (down to 4), not a single nematode is detected. Instead, a few copepod species numerically dominate this habitat (see [36]). The vigorous diffuse flow at the Siboglinid habitat (max. 32–54°C; max. 283 µM sulphide; min pH 4.4) shows very low diversity (S: 2±1; H′_loge_: 0.3±0.2). Bivalves with moderate diffuse flow (e.g. ∼8°C; ∼150 µM sulphide; ∼pH 6.7) are characterized by higher diversity (S: 9±2; H′_loge_ of 0.6±0.2) (Tables 5 and 6). Only evenness is similar (both J′: 0.6) in tube worm and bivalve habitats. Similar to seeps, often a single species highly dominates the vent community. On average, the dominance of a single species is 66%, but is ranging from 15 to 100%.

A possible reason of the low diversity and low evenness at vents is the fact that only a few species from the deep sea are adapted to the unfavourable environment, characterized by reduced chemical compounds (H_2_S and hydrocarbonates), or low oxygen concentrations. Interestingly, genera richness at vents is significantly lower than at seeps (Table 6). Possibly the extremely low genera richness at vents is explained by the fact that vents are more disturbed and stressed. Indeed, species present at deep-sea hydrothermal vents have to deal with volcanic eruptions, waxing and waning of vents and associated fluctuations of physico-chemical conditions (temperature, sulphide, oxygen, and pH) in relatively short time scales [4]. Seeps, on the contrary, are more long lived habitats, and only sometimes temperature or salinity anomalies are detected (see [5]). This apparently different physical nature of both chemosynthetic ecosystems is therefore likely to explain lower genera richness at vents. Other possible reasons are that the coexistence of nematodes, with various other, highly abundant epifaunal organisms at vents, might result in competitive exclusion, and could explain low eveness of single nematode species, in association with low densities. The extremly high abundant macrofauna at the studied vent sites could also predate on, or displace nematodes, causing high disturbance, and therefore keeping the habitat permanently in an early succession stage, with only a few nematode species surviving. Finally as explained for densities, substrate type could also have an influence on nematode diversity. All, except one of the nematode vent studies, concentrated on epifauna growing on hard substrates. Interestingly, epifaunal genera richness (mean S: 6) was significantly lower compared to infaunal genera richness (mean S: 18) from deep-sea chemosynthetic habitats (Tables 5 and 6). Also control bare basalts had a low nematode diversity, whilst in control sediments diversity was high. It will be interesting in the future to study nematodes from seep epifaunal communities and sediment vents in order to detect if substrate is a main driver of nematode richness at vents and seeps.

### Community similarity between seeps and vents

**Figure 3:**
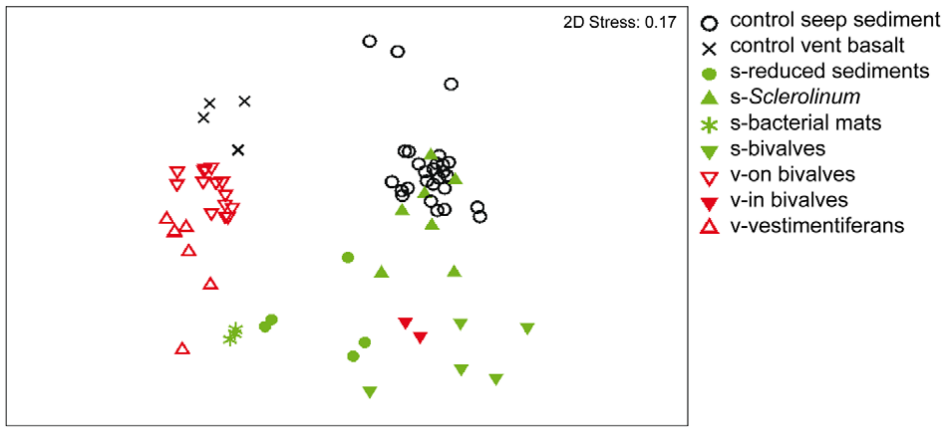
2-dimensional MDS configuration plot for 20 samples from deep-sea cold seeps (all in green colour), 22 samples from hydrothermal vents (all in red colour), and 27 samples from control samples (all in black). In addition to division into ecosystems by colour code, habitats of ecosystem are discriminated by different signs into (1) control seep sediment, (2) control vent basalt (both Control), (3) s-reduced sediments, (4) s-Siboglinid, (5) s-bacterial mats, (6) s-bivalves (s = seep; 3–6 all infauna), (7) v-on bivalves (v = vent, epifauna), (8) v-in bivalves (v = vent, infauna), and (9) vestimentiferans (v = vent, epifauna).

Vents and seeps are highly dissimilar (data used: [25]–[27] for seeps; [33], [35]–[39] for vents). They show on the average 91% dissimilarity as based on the Bray Curtis similarity index. Indeed, the MDS combined with ANOSIM significantly separates (R = 0.61; p = 0.001) the vent from the seep communities as illustrated in figure 3. Furthermore the seep fauna is more heterogeneous (only 21% similarity), compared to the vent communities (49% similarity), which cluster more tightly together in the MDS plot. Habitat types within the seep ecosytem show a very high heterogentity (dissimilarity always >63%, habitat similarity of single habitats 23–92%) (Table 5). Thus, these high community heterogenity at seeps can be explained by the different habitats sampled within the different seeps, including bacterial mats, reduced bare sediments, bivalve and Siboglinid habitats. The habitat heterogeneity within the vent samples is smaller, since most samples do come from bivalve aggregations (similarity 57%), although several tube worm samples (similarity 63%) were included too. The genus accounting most to similarity at vents is *Thalassomonhystera*, but is *Sabatieria* at seeps. *Thalassomonhystera* contributes with 16% to the high dissimilarity between vents and seeps.

Only the vent samples from the West Pacific near Fiji (loose sediment associated with bivalves) are outliers, in the sense that they do not cluster with the other vent samples, but rather with seep samples (close to seep bivalve infauna samples). Larger amounts of sediments, associated with the mussel samples collected at Fiji vents, were postulated earlier as responsible for these differences [33], pointing to the importance of substrate characteristics. Another interesting aspect is that the Siboglinid tube worm samples from seeps (mainly HMMV) fall totally within the range of control sediments in the MDS. The Siboglinid habitat was oxic and not sulphidic in the surface layers, allowing more regular deep-sea species to invade [25].

Control bare basalt samples cluster relativly close to the vent epifauna samples, but are 75% dissimilar (R = 0.75; p = 0.002). *Thalassomhystera*, being highly abundant at vents and low abundant on bare basalt, contributed with 10% most to the dissimilarity. In addition, members of Chromadorida, *Metoncholaimus*, and *Paracyatholaimus* were only found on bare basalt but not at vents. Control sediment samples cluster relatively close to the seep infauna samples (R = 0.23; p = 0.001), but also have a a high dissimilarity (78%). Genera causing the dissimilarity, were present in unequal densities in control and seep samples, with *Halomonhystera* and *Microlaimus* contributing with 5% each highest to the dissimilarity. This might point to the importance of substrate type (hard substrates at vents versus sediments in seeps) to nematode communities.

### Biogeographical distribution of seep and vent nematodes

Our knowledge on the biogeographical distribution of deep-sea seep and vent nematode genera and species is currently very limited. For vents, only data from the Pacific (North and South East Pacific Rise - EPR) [33]–[35], [38], [39], Guaymas Basin (GB) [40], West Pacific back-arc basins (WP, Fiji: [37]; Iheya ridge: [41]) and Atlantic (Mid-Atlantic Ridge - MAR) [38] are available. For seeps, only data from three sites of the Atlantic [25]–[27], and from one site in the Pacific Ocean (Sagami Bay: [30]) are published. No information on the Indian Ocean and Polar Regions is available yet.

**Figure 4:**
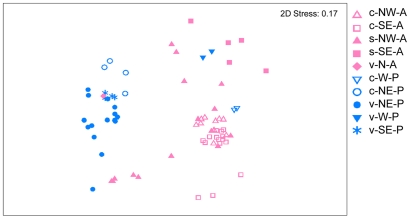
2-dimensional MDS configuration plot for deep-sea seep, vent, and control samples from the Atlantic (pink colour code) and from the Pacific (blue color code). Division into ecosystem type is given by letters (vent (v), seep (s), and control (c)) and filled signs (for v and s) and open signs (for c). Detailed biogeographical information is specified by letter codes: Atlantic (A), Pacific (P), North (N), West (W), NW (Northwest), SE (Southeast).

Multidimensional scaling of deep-sea seep, vent and control samples, based on nematode genera data, gives no evidence for distinction into large biogeographic provinces such as the Atlantic and the Pacific (Fig 4; data originally from [25]–[27], [33], [35]–[39,]. ANOSIM (with R = 0.52; p = 0.001) also shows that there is no major difference between samples from both oceans. However, dissimilarity is high (90%), but the Atlantic and the Pacific show themselves very low similarity, with only 26% and 32% respectively. Interestingly, hard substrate vent samples (with epifauna) from the North Atlantic (MAR) clusters with other hard substrate vent samples (and their controls) from the Eastern North and South Pacific (EPR). Sedimented vents and their controls (with infauna) from the West Pacific back arc basins cluster with various seep and control infauna samples from the Northwest and Southeast Atlantic. Therefore macro-ecological processes are at this stage of research more prominent than any biogeographical patterns, but the available data are too limited to draw conclusions.

Most seeps show taxonomically very different dominant species and genera, even at geographically adjacent sites (Nordic Margin) (Table 2). Striking is that many of the seep-dominant species (or at least morphologically similar species) are known from shallow water environments. *Halomonhystera disjuncta* for instance, the dominant species at the Barents sea slope, is a cosmopolitan, bacterivorous species known for its high resistance to environmental stress, especially to heavy metals [82]. *H. disjuncta* is further known as a fast colonizer in shallow waters, successfully exploiting organically enriched substrata, such as sediments from estuarine mudflats or decomposing algal thalli. The species occurs in shallow water along the European coast from both the North Sea, south of Norway [83] and the White Sea, northeast of Norway [84]. *H. disjuncta*, found at the Belgian and Dutch coast, is represented by at least 5 cryptic species [85] with subtle morphological differences [86]. Specimens collected at the Häkon Mosby Mud volcano (HMMV, Barents sea) were part of this species complex and closely related to shallow water specimens [26]. Through its record on the HMMV, it was the first time that *H. disjunta* is reported in high abundances at greater water depth, beyond the shelf. However the species was not found outside the bacterial mats or in any of the control sites, at least not in detectable quantities based on the applied sampling intensity.

At the Nyegga area (Atlantic Nordic margin) *Terschellingia* was one of the dominant genera in reduced black sediments, although represented with moderate to low densities (total: 288 ind. 10 cm^−2^, 36%: 104 ind. 10 cm^−2^) [26]. The specimens were identified as morphologically similar to *Terschellingia longicaudata*, although molecular evidence is lacking so far. Like *H. disjuncta*, *T. longicaudata* has an extensive geographical range including estuarine sediments off the North Sea [82], [83], [87], mangrove mudflats off northeastern Australia [88]–[90], the southeast coast of India [91], off the Atlantic coast of France, in the Black Sea [92] and the Gulf of Mexico, off eastern China (Qingdao province [93]) as well as New Zealand and the Solomon Islands [94]. In [95] a broad ecological and geographic range is suggested based on molecular evidence. However further evidence to confirm this, as also the importance of cryptic speciation in this species, is still required.

*Sabatieria mortenseni* is recently observed as the dominant species in the REGAB cold, seep adjacent to the Congo canyon in the Gulf of Guinea (South Atlantic [27]). *S. mortenseni* is again a cosmopolitan nematode species, known from littoral habitats with a high amount of clay and mud in Brazil, USA, Antarctica [96]–[98] and in the Strait of Magellan (Chile) in a depth range of 8–550 m [99]. The observation of a dominant species in the reduced seep sediments, known from shallow waters and with a wide distribution, is similar to the dominant nematode from the sulphide-rich sediments at Häkon Mosby Mud Volcano (*H. disjuncta* [25]). However again molecular evidence is lacking to support the suggested link between organically enriched shallow water fauna and deep-water seep fauna.

That seeps along continental margins show a past or ongoing connection with shallow water may seem odd at the first sight. However also other taxa show evidence of strong taxonomic simmilarities between seep and organically enriched shallow water fauna, such as the polychaete family Capitellidae. Indeed [20] already hypothesized that some seep invertebrate species may be derived from shallow-water species. Like wood for mussels [100], drifts of loose seaweed (Fucus sp.), as observed at the HMMV [26], may function as a possible transport medium for nematode species, on the condition that the exchange took place from the shallow-water to the deep-sea habitats. Moreover, observations of other species rafting on seaweed [85], suggest that nematodes have substantial dispersal capacities in shallow water, despite lacking pelagic larvae. However, the observations on the HMMV do not exclude the possibility of migration through stepping stones from the deep sea to shallow water. Further molecular investigations of the nematode communities at nearby intertidal and deep-sea habitats are required to resolve these issues.

Furthermore due to the absence of evidence for common dominant taxa (at species, genus or even family level) shared between geographically distinct seeps, it seems likely that shallow-deep water connections are stronger than deep-water dispersal routes. The lack of substantial deep-water tranpsort media like algae or wood may be the reason. However, it is too early to make statements on the limited dispersal between seeps, because of the low number of sites investigated so far.

At vents there seems a common (sub)dominance of the genus *Thalassomonhystera* at EPR, MAR and WP (Table 3). *Thalassomonhystera* appears to be a typically dominant bathyal genus, not only at hydrothermal vents and at the nearby basalt [36] but also in regular deep-sea sediments worldwide [3], [58], [101]–[103]. It belongs to the same family as the *Halomonhystera* found at the HMMV seep. Earlier morphological comparions of the *Monhystera* ( = *Thalassomonhystera*) species at the Fiji Basin, showed that the with *Bathymodiolus* associated species were different from the control site species. However undersampling may have biased the conclusions drawn [37]. However, a same *Thalassomonhystera* species (*T. fisheri*) was found at vents and on the nearby non-vent basalt at EPR [36]. Therefore the repeated high taxonomic similarity between the dominant vent species and the dominant control sediment species, suggests that local colonization and adaptation, rather than long distance dispersal seeds the vent fauna [30], [37], [39].

The similarity in nematode genus composition between vent ecosystems and control sites, stands in clear contrast with the presence of a specialized endemic hydrothermal megafauna. There is no specially adapated nematode fauna observed at vents so far. No symbionts are found, while the dominant vent genera are also present in the regular deep-sea sediments. The ephemeral nature of vents, in combination with their strongly isolated nature, possibly does not allow for the evolution and distribution of specially adapted forms, like in the macro- or megabenthic size class. Furthermore nematodes do lack pelagic life stages, and in absence of transport media like drifting algae, they are unable to cross long distances over relatively short time scales, like for larger hydrothermal animals [104]. The dispersion of nematodes in the deep sea seems much more dependent on the spatial continuity of the habitat on a relatively small scale on the one hand, and on their tolerance for variable environmental conditions on the other. It is therefore suggested, that for nematodes living in hydrothermal vents, invasion from adjacent sediments is potentially much more important than the distribution of a strongly specialized fauna over long distances.

It was already hypothesized before, that meiofaunal organisms living in thiobiotic conditions, were originally derived from oxybiotic species, which adapted to live in adjacent reduced environments [30], [44], [105]. The similar generic – but diverging species – composition between the hydrothermal vent and adjacent deep-sea sediments like in WP Fiji vents [37] possibly indicates that among oxic colonizers, certain taxa are less competitive in the usual oxic conditions, and are able to colonize the nearby sulphidic seeps successfully. Furthermore, the associations of oxybiotic species are often much more diverse, in terms of number of species than those in the thiobiotic communities. The latter are characterized by a decrease in congeneric species diversity and an increasing dominance [37], [44].

Not only Monhysteridae are found at vents. [40] collected meiofauna at hydrothermal sites at 21°N EPR and Explorer Ridge. The study mentioned a nematode community completely different from the community present in the normal deep-sea sediment, especially because of the presence of Epsilonematidea and Draconematidea in the hydrothermal meiofauna ecosystem. However, the Epsilonematidea seemed to be misidentified and appeared to be Desmodoridae [37]. Also in samples of sediments covered with bacteria from hydrothermal vents of GB [40] one new species (*Desmodora alberti* sp. nov.) was found, whereas *Desmodora marci* sp. nov. specimens were gathered in the WP Lau Basin (Hine Hina site, 1707 m, [106], [107]). Nematodes from the family Desmodoridae are present in many deep-sea nematode communities, but generally in low densities [3], [102]. *Desmodora* is also dominant in some Meteor seamount samples characterized by coarse biogenic sediments composed of corals and mollusc shells, and by strong current activity [3], [108], [109]. The combination of a higher tolerance for sulphidic environments and a preference for coarse substrates, likely makes this genus more successful at vents.

### Adaptations

Nematode genera from deep-sea hydrothermal vents and cold seeps have not developed any obvious adaptations, but they must have certain tolerance for sulphidic and/or anoxic conditions. For example, the *Halomonhystera disjuncta* species is thriving up to 5 cm depth in the sulphidic sediments of the *Beggiatoa* mats, but no evidence of detoxification mechanisms such as sulphur inclusions were found. Further, these individuals of the HMMV showed no remarkable morphological differences with the shallow-water specimens. Even the ovoviviparous reproduction mode of the HMMV species was also observed in the shallow water populations, although this characteristic was facultative and usually expressed in toxic environments [25]. Ovoviviparity is only known for a few marine nematode species. Permanent sulphidic sediments, in combination with anoxia at HMMV, create harsh conditions, which suggests that internal development of juveniles is an adaptation for securing the survival and growth of the vulnerable brood. Since brooding behaviour requires a substantial parental energy investment, it must provide strong benefits. The immediate motility of the new recruits allows migration in and out the anoxic and sulphidic sediments. It ensures the temporary availability of oxygen to both embryos and juveniles which is necessary for proper growth [110].

The genus *Terschellingia* is found in seeps, but also in muddy sediments rich in hydrogen sulphide, where it is known as a representative of the “thiobios” [111], [112]. *Terschellingia* is a typical inhabitant of the deeper sediment horizons [113]–[117] in these shallow water sediments. Thus, the genus might be overall tolerant for sulphidic and anoxic conditions. Also *Sabatieria* is typically present in enriched muddy sediments all over the world, and shows generally low abundances in sand [66], [102], [111], [112], [118]–[122]. Many of its species are considered eurytopic and tolerant of unstable, highly polluted environments [123]–[124]. *Sabatieria* is often the only remaining species in the most stressed situations, such as under high pollution pressure, or towards the centre of shallow, cold seeps [44], [55], [125]. *Sabatieria* dwells deep into the sediment, and is known to have its population maximum in the RPD [45], [113], [122], [126]. This points out to a preference for suboxic or anoxic environments, where a substantial fraction of the organic matter becomes incorporated below the oxic zone of the sediment [102]. However the exact mechanisms of adaptation of *Terschellingia* or *Sabatieria* species to the sulphidic or oxic environment remains unclear. Some authors pointed to the presence of dark, often multilayered intracellular globules in the intestinal cells of nematode species typical for sulphidic muds (i.e. *Sabatieria wieseri*, *Terschellingia longicaudata*, *Sphaerolaimus papillatus*, *Siphonolaimus ewensis*, *Pontonema vulgare*). However, their significance is ambiguous and their adaptive value for the thiobiotic life rather disputed [55], [127].

*Thalassomonhystera* is the most typical genus at deep-sea hydrothermal vents. However, this genus is also a regular member of normal deep-sea sediments [3], [58], [102]–[103]. Detailed studies on the physiological tolerance limits (i.e. temperature, sulphide, oxygen concentrations) of the genus in general, and of the species *T. fisheri* in particular are lacking. Also, no detailed morphological observations were done in order to detect if the species developed any special adaptations in the vent environment, i.e. such as sulphur inclusions. We speculate that, similar to genera found at seeps, also this genus has no special adaptations, but a broad ecological niche.

### Symbiosis

Although many of the macro-invertebrate taxa at seeps and vents harbour symbionts, hitherto none of the vent or seep nematode species show evidence of symbionts. Endo- and ectosymbioses with chemosynthetic bacteria do exist within the nematodes, but are mainly restricted to shallow-water habitats. The Stilbonematinae are a small group of marine free-living nematodes with sulphur-oxidizing ectosymbionts, who live in sheltered intertidal and subtidal marine, sulphide-rich sediments, where they migrate around the redox boundary depth [120], [128], or in shallow sublittoral hydrothermal vents [53], [56]. Stilbonematinae were only observed once, so far, from deep water at 900 m water depth in the NE Atlantic, with no evidence of active seeping [129]. Recently the nematode *Parastomonema* was found with higher numbers at some deep stations in the Whitard canyon (Ingels, personal communication), but again none of these sites showed evidence of seeping. *Parastomonema* is like *Astomonema* a mouthless and gutless nematode with endosymbiotic bacteria. *Astomonema southwardorum* (27%, Siphanolaimidae [130]) was the dominant species in a large pockmark with active methane seepage in the North Sea (153–167 m depth) [43], but is so far not recorded from deep-sea vents or seeps. *Desmodora masira* was found in the Oxygen Minimum Zone of the Santa Barbara basin with epicuticular, likely ecto-symbiotic, bacteria [131].

### Trophic interactions

Most nematodes from seeps and vents are classified as deposit feeders, based on their small buccal cavity and the absence of teeth. Typical deposit feeders are for example *Terschellingia*, *Sabatieria*, *Halomonhystera*, or *Thalassomonhystera*. At seeps, analyses of the fatty acids and stable isotopic signatures of the *Halomonhystera* species from HMMV [132] indicate that this species thrives on chemosynthetically derived carbon, as provided by the free-living sulphide-oxidizing bacteria. The digestive tract is fully developed in this monhysterid species, and there is no evidence for endo- or ectosymbionts as based on SEM or TEM observations. Also at vents, the large majority of nematodes are deposit feeders [39], however biomarker analysis are lacking so far. Only a few genera found at vents or seeps, such as the desmodorids or chromadorids have teeth in their buccal cavity. Interestingly, predators were so far never found abundant in deep-sea seeps or vents, altough they are a common part of the nematode community in many other ecosystems, including shallow water vents such as the *Oncholaimus* species in Mediterranean shallow vents [72].

### Comparison with shallow water seeps and vents

Shallow seeps are characterized by lower densities, compared to deep-water seeps. However, also at shallow water seeps high variations are the rule, and densities are ranging from 1 to 3070 ind. 10 cm^−2^ [29], [43], [46], [47], [50], [51,] (Table 4). Except for the dominance of a *Sabatieria* species and a monhysterid in two shallow seep studies, there is no further similarity in nematode fauna from shallow and deep waters.

Shallow vents are, similar to deep-water vents, characterized by low densities (often <100 ind. 10 cm^−2^), with values ranging from 1 to 1075 ind. 10 cm^−2^ [52]–[57]. There is no major nematode genus similarity between shallow and deep-water vents. Also studies on macro- and megafauna showed that the dominating fauna from shallow-water vents are different from those found at deep-sea vents [133]. Deep-sea vents are based on chemosynthetic production, whilst the co-presence of light and geothermal fluids at shallow vents promotes both photo- and chemosynthetic primary production, although this latter usually plays a secondary role [133]. This dualism complicates the identification of the different functional roles of components in these systems. The bulk of biomass in shallow water chemosynthetic ecosystems does not depend on symbiotrophs, but on organisms that feed on the available organic resources (i.e. deposit feeders, predators, omnivores). Previous studies on shallow vent nematodes (Table 4) provided conflicting results. In Paleohory Bay and Sulawesi (Indonesia) scavengers like *Oncholaimus* were dominant [56], [57], while in Kraternaya Bay the main trophic resource was represented by diatoms, thus leading to the dominance of epistrate (diatom) feeders among the polychaetes [134]. However, in Sulawesi, the nematode community was also dominated by epistrate feeders such as *Pomponema*, which were according to [57] favoured by the high primary biomass. The lack of symbiotic organisms and the presence of the genus *Oncholaimus*, which can also feed on ‘sulphur-bacteria’, suggest that the microbial biomasses in shallow vents still can represent an important food source capable of influencing the trophodynamics of these extreme system. Also here biomarker analysis is need to unravel the trophic links.

### Future perspectives

Seep and vent meiofauna remains largely unexplored, considering the low number of samples and geographical areas investigated, compared to the wide geographical distribution of the habitats. There is a strong need for more ecological, biogeographical and taxonomical research, using biomarker, molecular, physiological, and (ultra)morphological analysis, in order to understand their trophic position and importance, their origin and distribution and their adaptation. More specific the following aspects should be prioritized for future reseach on vents and seeps: (1) We can only speculate so far on the connectivity between isolated seeps and vents and the dispersal mechanisms for nematodes. Through a better taxonomical and biogeographical knowledge, nematodes can be used as model taxa for dispersal models for non-pelagic organisms within the deep-sea or between the shelf and slope. (2) Understanding the high capacity of nematodes to thrive in anoxic and/or sulphidic conditions may open a window to understand life in even more extreme conditions in past and future. The threat of large scale anoxia and ocean acidification through global warming urge the needs for identification of adaptational processes through micro-evolution and speciation on a relatively short time scale, for which vents and seeps can act as natural experiments. And (3) in order to understand the importance of competition, and other intra- and interspecific interactions in the colonization of vents and seeps , small scale *in situ* experiments by means of exclusion or transplantation can relatively easily be applied, through the availability of Remote operated technology. This way insight is gained on fundamental ecological processes and interactions in deep-sea vents and seeps.

### Conclusions

Although hydrothermal vents and cold seeps (both ecosystems based on chemosynthesis) have biochemical similarities, distinct differences are present in the standing stock, diversity and taxonomical composition of the meiofauna in both types of ecosystems.

Nematodes can benefit from the elevated in situ primary production at seeps, where they are often thriving in the sulphidic sediments, with standing stock values exceeding largely those at background sites. At vents, nematode densities and biomass are always low. It is likely that the presence of hard or soft substrates, the latter being more favourable for nematodes, is reponsible for the differential response between seeps and vents. Competition for food- or space with macro- or megafauna, or even the meiofauna such as copepods is possibly an additional factor responsible for the high variation at seeps and the lower numbers at vents, although evidence is lacking so far.

Deep-sea cold seeps and hydrothermal vents are both characterized by low nematode diversity and high dominance of single species. The low genera and species richness can be explained by the harsh physico-chemical conditions present in both ecosystems. There is no unique affinity with seeps or vents at genus level, suggesting a lower taxonomic level of endemicity for nematodes compared with mega- and macrofauna. Dominant nematode genera varied among the different seeps and hydrothermal vents. Also between multiple cold seeps or hydrothermal vents are distinct taxonomical differences present.

Seep habitats are often densely populated by a single or a few species, belonging to generalistic genera, and often known from shallow water. It appears that chemosynthetic sediments, strongly affected by reduced fluids, generate a habitat that is difficult to exploit by most of the typical deep-sea nematode taxa, since the dominant nematode fauna from seeps differs significantly from the regular deep-sea fauna. Shallow water relatives, rather than typical deep-sea taxa have successfully colonized the reduced sediments at large water depth. However, if further molecular evidence confirms this connection, the exchange between the deep sea and the shallow water seems to have taken place several times since different shallow water taxa colonized geographically separated seeps. Vent communities are often dominated by *Thalassomonhystera*, a typical deep-sea genus. At vents the taxonomic simmilarity with adjacent regular deep-sea sediments is much higher, supporting rather the importance of local adaptation than that of long distance distribution. Likely the ephemeral nature of vents, its long distance offshore and the absence of pelagic transport mechanisms, has prevented so far the establishment of a successful and typical vent fauna. However the geographical coverage of both vent and seep studies at lower taxonomic level is too low until today.

Deep-sea hydrothermal vent or cold seeps communities appear to have no strong or exclusive affinity to other communities from sulphidic environments, such as the “thiobios” of sulphidic sediments, shallow-water vents, or cold seeps, despite the presence of reducing chemicals and hypoxia. Some thiobiotic genera, like *Sabatieria* and *Terschellingia* are shared, but not any of the known chemosynthetic nematode species with symbionts was found so far in deep-sea vents or seeps

The knowledge we have to day on deep-water seep and vent meiofauna is only a tip of the iceberg. By providing some future perspectives in meiofauna research, we hope that ecological research programs from now on, will include systematically the meiofauna in order to get an integrated picture of vent and seep biological processes.
